# EARLY PHYSICAL AND MENTAL HEALTH PREDICTORS OF NIGHTTIME DRIVING DIFFICULTY IN OLDER ADULTS

**DOI:** 10.1093/geroni/igac059.2322

**Published:** 2022-12-20

**Authors:** Colleen Peterson, Toben F Nelson

**Affiliations:** University of Michigan Transportation Research Institute, Ann Arbor, Michigan, United States; University of Minnesota, Minneapolis, Minnesota, United States

## Abstract

Older drivers are expected to make up about 25% of licensed U.S. drivers by 2050. Describing early predictors of driving difficulty can identify interventions that might delay driving cessation or support successful transition to driving retirement. In this exploratory study, we analyzed nationally representative data from the longitudinal National Social Life, Health, and Aging Project (NSHAP) conducted in 2005–06, 2010-11, and 2015-16. Our purpose was to ascertain mental health and physical functioning correlates of increased nighttime driving difficulty, which often precedes daytime difficulties. The sample included 1,454 drivers with observations at all 3 waves aged 57 to 85 at baseline. By wave three, adjusting for non-response and sampling bias, 59% reported no driving difficulty, 23% reported some or much difficulty, and 17% reported no longer being able to drive at night. Results of mixed-effect ordinal logistic models, adjusting for demographics, showed increased inability to drive at night was associated most strongly with self-report of poor or fair physical health (OR=10.4, 95%CI=6.2,17.5; OR=2.4, 95%CI=1.8,3.2), considering oneself “not at all active” (OR=2.9, 95%CI=1.3, 6.4), and being unable to balance heel-to-toe for 10 seconds (unsuccessful attempt: OR=4.8,95%CI=1.5,15.5; less than 10 seconds: OR=2.3, 95%CI=1.1,4.9). Although generalized self-reported mental health was not associated with increased nighttime driving difficulty, each additional depressive symptom (CESD-11) increased the odds of having greater driving difficulty by 12% (95%CI=1.1, 1.5). This study found early predictors of increased nighttime driving difficulty and retirement, providing opportunity to inform interventions to reduce roadway risk to older adults and others.

